# Aktueller Wissensstand von Patient:innen über den perioperativen Einfluss einer Anämie und ihrer Ursachen

**DOI:** 10.1007/s00101-024-01498-y

**Published:** 2025-01-31

**Authors:** Janna Mock, Lotta Hof, Theresa Dhein, Esther Pollok, Vanessa Neef, Jochen Kaiser, Kai Zacharowski, Patrick Meybohm, Suma Choorapoikayil

**Affiliations:** 1 Klinik für Anästhesiologie, Intensivmedizin und Schmerztherapie, Universitätsklinikum Frankfurt, Frankfurt, Deutschland; 2 Institut für Medizinische Psychologie, Fachbereich Medizin, Goethe Universität Frankfurt, Frankfurt, Deutschland; 3 Klinik und Poliklinik für Anästhesiologie, Intensivmedizin, Notfallmedizin und Schmerztherapie, Universitätsklinikum Würzburg, Würzburg, Deutschland

**Keywords:** Patient:innen Befragung, „Patient empowerment“, „Patient Blood Management“, Eisenmangel, Operation, Patient survey, Patient empowerment, Patient Blood Management, Iron deficiency, Surgery

## Abstract

**Hintergrund:**

Trotz der guten Behandelbarkeit einer Anämie ist ihre Prävalenz mit 30 % in der Allgemeinbevölkerung und mit 35 % bei chirurgischen Patient:innen hoch. Symptome werden oftmals falsch gedeutet und die Anämie von Patient:innen nicht als Erkrankung wahrgenommen.

**Ziel der Arbeit:**

Ziel der vorliegenden Untersuchung war es, den Wissensstand von Patient:innen hinsichtlich einer Anämie bei bevorstehenden Operationen mit erhöhtem Blutverlust zu erheben.

**Material und Methoden:**

Hierzu wurden Patient:innen, die sich einer Operation mit einer Transfusionswahrscheinlichkeit > 10 % unterzogen, in der Prämedikationsambulanz der Klinik für Anästhesiologie, Intensivmedizin und Schmerztherapie des Universitätsklinikums Frankfurt jeweils im Mai 2023 und Oktober 2023 mithilfe eines Fragebogens befragt.

**Ergebnisse:**

Insgesamt konnten 196 ausgefüllte Fragebogen von Patient:innen ausgewertet werden. Die meisten korrekten Antworten wurden mit 54,3 % (*n* = 426/784) in der Kategorie „Anämiebehandlung“ gegeben, gefolgt von 50,1 % (*n* = 393/784) korrekten Antworten in der Kategorie „Anämiediagnostik“ und 48,8 % (*n* = 478/980) in der Kategorie „Perioperativer Einfluss einer Anämie“ sowie 44,6 % (*n* = 350/784) korrekten Antworten in der Kategorie „Einfluss der Ernährung“. Das größte Wissensdefizit wiesen die Befragten in der Kategorie „Anämiesymptome“ mit 38,1 % (*n* = 598/1568) korrekten Antworten und „Anämieursachen“ mit 36,1 % (*n* = 354/980) auf. Ein Großteil der Befragten (71,4 %; *n* = 140/195) hatte Interesse, mehr über die Anämien zu erfahren.

**Schlussfolgerung:**

Zusammenfassend konnte festgestellt werden, dass ein ausgeprägtes Wissensdefizit bei chirurgischen Patient:innen hinsichtlich einer Anämie vorliegt. Vor allem im Bereich der Ursachen und der Symptome einer Anämie wurde nur etwa ein Drittel der Aussagen korrekt beantwortet.

**Zusatzmaterial online:**

Die Online-Version dieses Beitrags (10.1007/s00101-024-01498-y) enthält das im Manuskript erwähnte Zusatzmaterial. Bitte scannen Sie den QR-Code.

## Abstract (en)

**Background:**

Despite being treatable, the prevalence of anemia is relatively high, affecting up to 30% of the general population and 35% of patients undergoing surgery. Symptoms are often misinterpreted and patients frequently do not recognize anemia as a disease. As a result, it is often not discussed during medical consultations. Additionally, the limited time between diagnosis and surgery makes preoperative anemia management challenging.

**Objective:**

The aim of the present study was to assess the state of knowledge about anemia and to identify areas of deficits to develop a strategy for future improvement.

**Material and methods:**

Patients undergoing surgery with a transfusion probability of > 10% were interviewed at the department for anesthesiology, intensive care medicine and pain therapy of the University Hospital Frankfurt in May and October 2023.

**Results:**

A total of 196 completed questionnaires were evaluated. Most correct answers were given in the category “treatment of anemia” with 54.3% (*n* = 426/784), followed by 50.1% (*n* = 393/784) correct answers in the category “diagnosis of anemia”, 48.8% (*n* = 478/980) in the category “perioperative influence of anemia” and 44.6% (*n* = 350/784) correct answers in the category “influence of nutrition”. The patients showed the greatest knowledge deficits in the category “symptoms of anemia” with 38.1% (*n* = 598/1568) correct answers and “causes of anemia” with 36.1% (*n* = 354/980). Only 3% (*n* = 5/196) of the participants rated their knowledge as “good”, 16% (*n* = 31/196) as “fairly good”, 48% (*n* = 94/196) as “fairly poor” and 29% (*n* = 56/196) as “poor”. There is a weak to medium positive linear correlation between the self-assessment and the correct answers given (correlation coefficient 0.44). Approximately 70% of participants had a higher level of education, such as vocational training, a university degree or a high school diploma. There was no correlation between the number of correct answers given and the participants’ level of education. The majority of the patients (71.4%; *n* = 140/195) expressed interest in learning more about anemia.

**Conclusion:**

We identified a significant knowledge deficit, particularly regarding the causes and symptoms of anemia. It is likely that knowledge gaps persist and that anemia does not receive sufficient attention in everyday clinical practice. Educating and training patients about anemia is essential to further reduce its prevalence.

## Kurze Hinführung zum Thema

Trotz der Tatsache, dass das Patient Blood Management mittlerweile in die Querschnitts-Leitlinie Hämotherapie der Bundesärztekammer im Jahr 2020 aufgenommen wurde, ist die Behandlung der präoperativen Anämie noch unzureichend. Aufgrund der oftmals begrenzten Zeit zwischen Vorstellung und Operation gestaltet sich das präoperative Anämiemanagement schwierig. Patient:innen können meist ihre Anämiesymptome nicht richtig zuordnen und thematisieren diese oft nicht im ärztlichen Gespräch. Das Ziel der vorliegenden Studie war es, den Wissensstand von Patient:innen bezüglich einer Anämie zu erheben.

## Einleitung

Im Allgemeinen beschreibt eine Anämie eine Reduktion der Zahl von Erythrozyten bzw. des Hämoglobingehalts. Die Symptome, unter denen die Patient:innen leiden, variieren von Symptomlosigkeit bis zu einer massiven Beeinträchtigung im Alltag durch z. B. Angina-pectoris-Beschwerden, verminderter Leistungsfähigkeit, Kopfschmerzen, Schwindel oder Dyspnoe [1]. Darüber hinaus ist eine präoperative Anämie ein unabhängiger Risikofaktor für eine erhöhte 30-Tage-Mortalität und Morbidität bei chirurgischen Patienten:innen [2]. Ein längerer Intensivstations- und Krankenhausaufenthalt sowie die vermehrte Gabe von Erythrozytenkonzentraten (EK) können die Folgen einer bestehenden präoperativen Anämie sein [3].

Die Prävalenz der Anämie beträgt weltweit ca. 30 %, bei chirurgischen Patient:innen wird sie mit bis zu 35 % angegeben [4]. Somit ist ungefähr jede dritte chirurgische Patient:in von einer Anämie betroffen. Die häufigste Ursache stellt die Eisenmangelanämie dar [3]. Andere Ursache können ein Vitamin‑B_12_- bzw. Folsäuremangel, eine chronische Erkrankung, wie z. B. eine Niereninsuffizienz, oder auch eine Tumorerkrankung sein [5]. Um den Wissensstand der Patient:innen über Anämie und die assoziierten Risiken einer präoperativen Anämie zu ermitteln, wurde eine Befragung in der Prämedikationsambulanz des Universitätsklinikums Frankfurt am Main durchgeführt.

## Material und Methoden

Im Rahmen der Implementierung des Patient Blood Management bei chirurgischen Patient:innen wurde eine Anämie-Ambulanz zu Diagnose und Therapie einer präoperativen Anämie etabliert (ClinicalTrials.gov, NCT02147795). Die Anämie-Ambulanz ist an die Prämedikationsambulanz der Klinik für Anästhesiologie, Intensivmedizin und Schmerztherapie des Universitätsklinikums Frankfurt am Main angegliedert, wo letztlich auch die Patient:innen-Befragung durchgeführt wurde. Die Studie wurde von der Ethikkommission des Universitätsklinikums Frankfurt (Ref. 318/17) und dem Hessischen Datenschutzamt (Ref: 43.60; 60.01.21-ga) genehmigt. Die Teilnahme der Patient:innen erfolgte auf freiwilliger Basis. Es wurde nicht erhoben, wie viele Patient:innen sich gegen die Teilnahme entschieden. Die Daten wurden anonymisiert erhoben. Es wurden keine Informationen, wie z. B. Hämoglobinwerte, aus der Krankenakte entnommen. Eine schriftliche Einwilligung der Patient:innen war nicht erforderlich.

Patient:innen wurden jeweils im Mai 2023 und im Oktober 2023 befragt. Es wurden 2 Monate für die Befragung gewählt, um mögliche saisonale Effekte zu vermeiden.

Im Rahmen des Prämedikationsgespräches wurden Patient:innen (≥ 18 Jahre) mit einer bevorstehenden Operation, die mit einer Transfusionswahrscheinlichkeit > 10 % einhergehen kann, befragt. Zu diesen Operationen gehören v. a. große tumorchirurgische Eingriffe in der Mund-Kiefer-Gesichtschirurgie, Urologie, Viszeral- und Thoraxchirurgie; herzchirurgische Eingriffe, gefäßchirurgische Eingriffe an der Aorta und Wirbelsäulenoperationen oder Endoprothetikeingriffe in der Unfallchirurgie und Orthopädie (Zusatzmaterial online: „Aufstellung Kliniken mit Indexoperationen“). Diese Patient:innen haben aufgrund ihrer anstehenden Operation ein höheres postoperatives Risiko für ein negatives Outcome im Vergleich zu Patient:innen mit einer Operation, die nur ein geringes oder kein Transfusionsrisiko aufweist. Für die Patientengruppe, die durch ihre Operation ein erhöhtes Transfusionspotenzial hat, ist eine Anämie in Bezug auf das perioperative Procedere besonders relevant. Patient:innen sollten ausreichende Deutschkenntnisse besitzen, um den Fragebogen allein auszufüllen.

Der selbst konzipierte Fragebogen bestand aus 17 „Multiple-choice“-Fragen und umfasste Wissensabfragen bezüglich einer Anämie zu folgenden Unterpunkten: Ursachen, Diagnostik, Symptome, perioperativer Einfluss, Behandlungsmöglichkeiten und Ernährung. Es sollten jeweils 4 bis 5 „Wahr- und Falschaussagen“ mit „richtig, falsch oder ich weiß nicht“ bewertet werden (Zusatzmaterial online: „Fragebogen zur Anämieaufklärung“). Hinsichtlich möglicher Unklarheiten bezüglich der Fragestellungen wurden die Fragen von einem Psychologen überprüft. Ein Zeitlimit wurde nicht festgelegt; Patient:innen sollten den Fragebogen selbstständig ausfüllen.

Die Selbsteinschätzung der Befragten bezüglich ihres Wissens über die Anämie wurde in 4 Kategorien eingeteilt und ausgewertet: „gut“ (100–76 %), „eher gut“ (75–51 %), „eher schlecht“ (50–26 %) und „schlecht“ (< 25 %). Eine mögliche Korrelation zwischen Selbsteinschätzung und Bildungsstand wurde mittels Pearson ermittelt. Als höherer Bildungsabschluss wurden eine abgeschlossene Berufsausbildung, (Fach‑)Hochschulabschluss und (Fach‑)Abitur zusammengefasst.

Die Antworten wurden in eine Excel-Tabelle übertragen und ausgewertet.

## Ergebnisse

### Patientencharakteristika

Insgesamt wurden im Mai (*n* = 113/196) und im Oktober (*n* = 83/196) 2023 196 Patient:innen befragt. Dabei waren 73,5 % (*n* = 144/196) der Befragten männlich, 24,5 % (*n* = 48/196) weiblich und 2,0 % (*n* = 4/196) ohne Angaben. Die meisten Teilnehmer:innen (31,6 % (*n* = 62/196)) waren zwischen 61 und 70 Jahre alt, gefolgt von 25,5 % (*n* = 50/196) in der Altersspanne von 71 bis 80 Jahren, 22,4 % (*n* = 44/196) zwischen 41 und 50 Jahren sowie geringen Anteilen in den Altersspannen von 18 bis 30 Jahren (3,6 % (*n* = 7/196)) und 31 bis 40 Jahren (1 % (*n* = 2/196)). Die über 80-Jährigen waren mit 11,2 % (*n* = 22/196) vertreten.

Ein Großteil der Patient:innen (54,6 %, *n* = 107/196) war aus der Klinik für Herz- und Gefäßchirurgie, 18,9 % (*n* = 37/196) kamen aus der Allgemein‑, Viszeral‑, Transplantations- und Thoraxchirurgie und 16,8 % (*n* = 33/196) aus in der Urologie. Der Rest entfiel auf Patient:innen der Mund-Kiefer-Gesichtschirurgie (2,0 %; *n* = 4/196), Unfallchirurgie (1,0 %; *n* = 2/196) und Patient:innen, die mehr als eine Fachabteilung nannten (1,5 %; *n* = 3/196). Die Angabe „Sonstiges“ wurde von 4,6 % (*n* = 9/196) verwendet.

Es gaben 86,7 % (*n* = 170/196) der Befragten an, dass für sie kürzlich ein Blutbild angefertigt wurde. Bei 85,7 % (*n* = 168/196) wurde bisher noch keine Anämie festgestellt. Insgesamt gaben 83,7 % (*n* = 164/196) der Teilnehmer:innen an, dass kein Familienmitglied oder Freund unter einer Anämie leiden würde. Die Mehrheit (91,3 %; *n* = 179/196) der Befragten war zum Zeitpunkt der Befragung noch nicht in der Anämie-Ambulanz vorstellig, bei 81,1 % (*n* = 159/196) sei auch kein Besuch mehr geplant, oder sie gaben an, es nicht zu wissen (11,2 %; *n* = 22/196) (Zusatzmaterial online: „Patientenbezug zur Anämie“).

Um einen groben Überblick über die korrekt gegebenen Antworten zu erhalten, kann man diese zunächst kategorienübergreifend analysieren. In dem Bereich von 100–76 % der korrekt gegebenen Antworten konnte man insgesamt 8 % (*n* = 16/196) der Teilnehmer:innen verzeichnen. Insgesamt 42 % (*n* = 83/196) der Befragten befanden sich im Bereich 75–51 % korrekter Antworten. In der Spannweite von 50–26 % der korrekt gegebenen Antworten befanden sich 27 % (*n* = 52/196) der Teilnehmer:innen. Weniger als 25 % korrekte Antworten gaben 22 % (*n* = 43/196) der Patient:innen.

Etwa ein Drittel der Befragten gab als höchsten Bildungsabschluss eine abgeschlossene Berufsausbildung (32,1 % (*n* = 63/196)) oder einen (Fach‑)Hochschulabschluss (30,1 % (*n* = 59/196)) an. Für 7 % (*n* = 15/196) der Befragten war das (Fach‑)Abitur der höchste Bildungsabschluss. Einen Hauptschulabschluss wiesen 10,2 % (*n* = 20/196) und einen Realschulabschluss 13,8 % (*n* = 27/196) der Teilnehmer:innen auf. Nur eine Minderheit von 1,5 % (*n* = 3/196) hatte keinen Abschluss. Fasst man eine abgeschlossene Berufsausbildung, (Fach‑)Hochschulabschluss und (Fach‑)Abitur als einen höheren Bildungsabschluss zusammen, wiesen diesen in etwa 70 % der Befragten auf. Betrachtet man den Zusammenhang zwischen Bildungsabschluss und korrekt gegebenen Antworten, kann man feststellen, dass in dem Bereich von 100–76 % der korrekt gegebenen Antworten 75 % (*n* = 12/196) der Teilnehmer:innen einen höheren Bildungsabschluss hatten. Bei 75–51 % der korrekt gegebenen Antworten wiesen 63 % (*n* = 52/196) der Teilnehmer:innen einen höheren Bildungsabschluss auf. Auch unter den Teilnehmer:innen mit 50–26 % der korrekt gegebenen Antworten wiesen 77 % (*n* = 40/196) einen höheren Bildungsabschluss auf. Bei weniger als 25 % der korrekt angegebenen Antworten, hatten 72 % (*n* = 31/196) einen höheren Bildungsabschluss. Es besteht keine Korrelation zwischen den korrekt angegebenen Antworten und dem Bildungsstand der Teilnehmer:innen (Korrelationskoeffizient 0,06–0,08).

### Wissenstand der Patienten zu Anämie

**Abb. 1:**
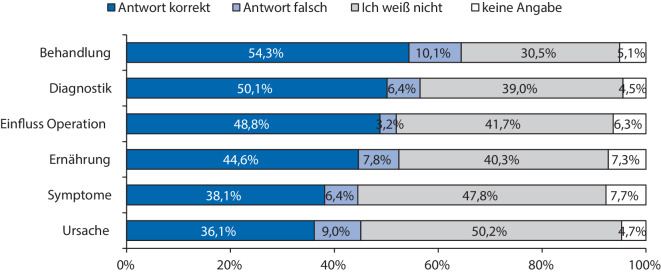
Übersicht über den Anteil der korrekten Antworten gegenüber falschen Antworten, der Antwort „ich weiß nicht“ und keinen Angaben in den einzelnen Kategorien

In der Kategorie „Ursache einer Anämie“ und „Symptome einer Anämie“ wurden mit 36,1 % (*n* = 354/980) und 38,1 % (*n* = 598/1568) die wenigsten richtigen Antworten gegeben (Abb. 1).

**Abb. 2:**
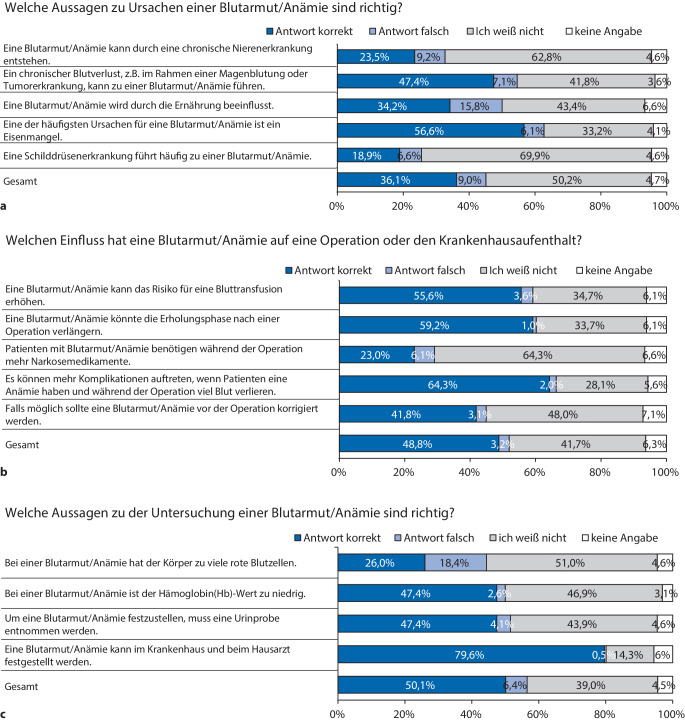
Detaillierte Übersicht über die Kategorien „Ursachen einer Anämie“, „Perioperativer Einfluss einer Anämie“ und „Diagnostik einer Anämie“

Etwa der Hälfte der Patient:innen war bekannt, dass ein Eisenmangel (56,6 % (*n* = 111/196) korrekte Antworten) oder ein chronischer Blutverlust (47,4 % (*n* = 93/196) korrekte Antworten) die Ursache für eine Anämie sein können. Seltener war bewusst, dass eine Anämie durch eine chronische Nierenerkrankung (23,5 % (*n* = 46/196) korrekte Antworten) verursacht oder durch die Ernährung beeinflusst werden kann (34,2 % (*n* = 67/196) korrekte Antworten). Nur 18,9 % (*n* = 37/196) aller Befragten markierten die Aussage „Eine Schilddrüsenerkrankung führt zu einer Blutarmut/Anämie“ korrekt als falsch (Abb. 2a).

**Abb. 3:**
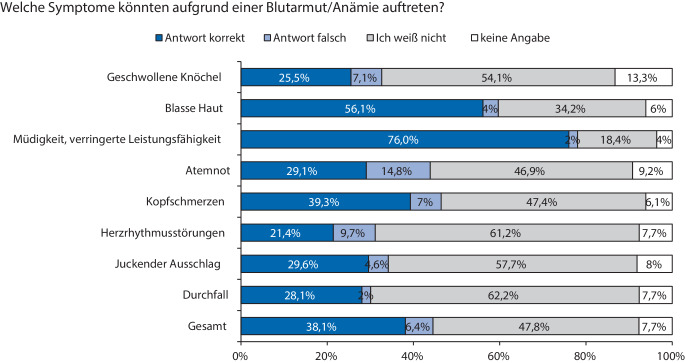
Detaillierte Übersicht zu „Symptomen einer Anämie“

In der Kategorie „Symptome einer Anämie“ konnten 38,1 % (*n* = 598/1568) der Patient:innen mögliche Symptome einer Anämie zuordnen. Müdigkeit und eine verminderte Leistungsfähigkeit erkannten 76,0 % (*n* = 149/196) der Befragten als typische Symptome einer Anämie. Zusätzlich kreuzten 56,1 % (*n* = 110/196) an, dass auch eine blasse Haut auf eine Anämie zurückzuführen sein könnte. Weniger war bekannt, dass eine Anämie für Kopfschmerzen (39,3 % (*n* = 77/196) korrekte Antworten), Atemnot (29,1 % (*n* = 57/196) korrekte Antworten) und Herzrhythmusstörungen (21,4 % (*n* = 42/196) korrekte Antwort) ursächlich sein kann. Aussagen, dass geschwollene Knöchel (25,5 % (*n* = 50/196) korrekte Antworten), juckender Ausschlag (29,6 % (*n* = 58/196) korrekte Antworten) und Durchfall (28,1 % (*n* = 55/196) korrekte Antworten) Symptome eine Anämie sein können, wurden nur selten als Falschaussage markiert (Abb. 3).

In der Kategorie „Einfluss der Ernährung“ haben 44,6 % (*n* = 350/784) der Patient:innen die gestellten Fragen richtig beantwortet. Rotes Fleisch wurde von 58,7 % (*n* = 115/196), Nüsse von 55,1 % (*n* = 108/196) und Linsen von 45,9 % (*n* = 90/196) der Befragten korrekt als gute Eisenlieferanten bewertet. Nur in etwa einem Fünftel (18,9 % (*n* = 37/196)) war bewusst, dass Äpfel nicht zu den guten Eisenlieferanten zählen (Abbildung „Einfluss der Ernährung“ im Zusatzmaterial online: „Wissensstand“).

In der Kategorie „Perioperativer Einfluss einer Anämie“ wurden 48,8 % (*n* = 478/980) der Aussagen korrekt angekreuzt. Insgesamt 64,3 % (*n* = 126/196) der Befragten bejahten die Aussage, dass mehr Komplikationen auftreten können, wenn Patient:innen unter einer Anämie leiden und während der Operation einen höheren Blutverlust verzeichnet wird. Dass eine Anämie die Erholungsphase nach einer Operation verlängern kann, kreuzten 59,2 % (*n* = 116) der Befragten an. Nur in etwa die Hälfte der Befragten (55,6 % (*n* = 109/196) korrekte Antworten) wusste, dass eine Anämie das Risiko für eine Bluttransfusion erhöhen kann. Weniger Befragten war bewusst, dass eine Anämie am besten vor einer Operation korrigiert werden sollte (41,8 % (*n* = 82/196) korrekte Antworten) (Abb. 2b).

In der Kategorie „Diagnostik einer Anämie“ wurden 50,1 % (*n* = 393/784) der Aussagen korrekt beantwortet. Die meisten Patient:innen wussten, dass eine Anämie beim Hausarzt sowie im Krankenhaus festgestellt werden kann (79,6 % (*n* = 156/196) korrekte Antworten). Seltener wurde korrekt erkannt, dass der Hämoglobinwert bei einer Anämie zu niedrig ist (47,4 % (*n* = 93/196) korrekte Antworten) (Abb. 2c).

Die meisten korrekten Antworten wurden in der Kategorie „Behandlung einer Anämie“ gegeben (54,3 %, *n* = 426/784). Insgesamt 71,4 % (*n* = 140/196) der Befragten wussten, dass zur Behandlung einer Eisenmangelanämie Eisen als Tablette eingenommen oder als Infusion verabreicht werden kann. 67,3 % (*n* = 132/196) der Befragten kreuzten die Aussage, dass eine Anämie nicht nur bei Frauen behandelt werden kann, als korrekt an. Der Mehrheit war zudem bewusst, dass ernährungsbedingte Anämien in der Regel gut behandelbar sind (67,3 % (*n* = 132/196)). Dass regelmäßiger Sport das Risiko für eine Anämie senken kann, kreuzten nur 11,2 % (*n* = 22/196) als Falschaussage an (Abbildung „Behandlung einer Anämie“ im Zusatzmaterial online: „Wissensstand“).

Nur 3 % (*n* = 5/196) der Befragten schätzten ihr Wissen als „gut“ ein, 16 % (*n* = 31/196) als „eher gut“, 48 % (*n* = 94/196) als „eher schlecht“ und 29 % (*n* = 56/196) als „schlecht“ ein. Vergleicht man die Selbsteinschätzung mit den korrekt gegebenen Antworten, haben sich insgesamt 36 % der Patient:innen richtig eingeschätzt. Es besteht ein schwach bis mittelstark positiver linearer Zusammenhang zwischen der Selbsteinschätzung und den korrekt gegebenen Antworten (Korrelationskoeffizient 0,44).

Etwa ein Drittel der Befragten gab als höchsten Bildungsabschluss eine abgeschlossene Berufsausbildung (32,1 % (*n* = 63/196)) oder einen (Fach‑)Hochschulabschluss (30,1 % (*n* = 59/196)) an. Einen Realschulabschluss besaßen 13,8 % (*n* = 27/196), 10,2 % (*n* = 20/196) einen Hauptschulabschluss und 7 % (*n* = 15/196) ein (Fach‑)Abitur. Nur eine Minderheit hatte keinen Abschluss (1,5 % (*n* = 3/196)). Fasst man eine abgeschlossene Berufsausbildung, (Fach‑)Hochschulabschluss und (Fach‑)Abitur als einen höheren Bildungsabschluss zusammen, wiesen diesen in etwa 70 % der Befragten auf. Betrachtet man den Zusammenhang zwischen Bildungsabschluss und korrekt gegebenen Antworten, kann man feststellen, dass in dem Bereich von 100–76 % der korrekt gegebenen Antworten 75 % (*n* = 12/196) der Teilnehmer:innen einen höheren Bildungsabschluss hatten. Bei 75–51 % der korrekt gegebenen Antworten wiesen 63 % (*n* = 52/196) der Teilnehmer:innen einen höheren Bildungsabschluss auf. Auch unter den Teilnehmer:innen mit 50–26 % der korrekt gegebenen Antworten wiesen 77 % (*n* = 40/196) einen höheren Bildungsabschluss auf. Bei weniger als 25 % der korrekt angegebenen Antworten, hatten 72 % (*n* = 31/196) einen höheren Bildungsabschluss. Es besteht keine Korrelation zwischen den korrekt angegebenen Antworten und dem Bildungsstand der Teilnehmer:innen (Korrelationskoeffizient 0,06–0,08).

Ein Großteil der Befragten (71,4 %; *n* = 140) hatte Interesse, mehr über die Anämien zu erfahren.

## Diskussion

Die präoperative Anämie, die mit einer Prävalenz von 35 % auftritt, ist ein modifizierbarer Risikofaktor [6]. Es wurde mehrfach belegt, dass ein präoperatives Anämiemanagement, insbesondere die Therapie von Eisenmangelanämien, das Outcome chirurgischer Patient:innen verbessert [7]. Dennoch ist ein umfangreiches Anämiemanagement sowohl im klinischen Setting, aber v. a. auch im ambulanten Bereich, noch unzureichend umgesetzt. Gründe hierfür sind u. a. eingeschränkte finanzielle Ressourcen, die schlechte Planbarkeit und das geringe Bewusstsein der Patient:innen, aber auch der Ärzt:innen [8]. Immer mehr Studien propagieren eine Beteiligung der Patient:innen an ihrer Behandlung, dazu gehören das Verständnis und das Akzeptieren der Erkrankung [9, 10].

In der vorliegenden Studie wurde der Wissensstand zum Thema Anämie von 196 chirurgischen Patient:innen erfragt. Verwendet wurde ein selbst konzipierter Fragebogen, der uns als Hilfestellung für einen zukünftigen Informationsflyer über eine Anämie dienen sollte. Aus diesem Grund verwendeten wir eine Reihe von Aussagen, die wir unsererseits als wichtig erachteten, die mit „richtig“, „falsch“ oder „ich weiß nicht“ beantworten werden sollten. Diese Auswahlmöglichkeiten könnten die Patient:innen in eine Richtung lenken. Auf Freitextantworten verzichteten wir jedoch bewusst, da dies ein höheres Patientenengagement, das oft im klinischen Setting bei den abzuarbeitenden Punkten nicht immer gegeben ist, vorausgesetzt hätte. In zukünftigen Studien sollte man die Fragen neutraler und offener formulieren, um eine mögliche Beeinflussung zu vermeiden. Unseres Wissens nach gibt es bisher keinen validierten Fragebogen zur Erhebung des Wissensstands einer Anämie im chirurgischen Kontext.

Die Ursachen einer Anämie können multifaktoriell sein und z. B. durch eine Mangelernährung, eine chronische Blutung, eine chronische Nierenerkrankung, eine Tumorerkrankung oder eine verstärkte Menstruation hervorgerufen werden [5]. Obwohl viele ältere Patient:innen, die sich einem größeren chirurgischen Eingriff unterziehen, an einer chronischen Nierenerkrankung oder unter einem chronischen Blutverlust leiden, wusste etwa ein Drittel der Befragten nicht, dass diese Vorerkrankungen ursächlich für eine Anämie sein können [11]. Ein möglicher Kritikpunkt könnte sein, dass Patient:innen die Formulierungen chronische Nierenerkrankung oder chronischer Blutverlust nicht ausreichend verstehen und es aus diesem Grund zu einer reduzierten Anzahl an korrekten Antworten gekommen sein könnte. Auch bei dem Abschnitt „Diagnostik“ verwendeten wir den Fachbegriff Hämoglobinwert. Auch hier könnte die Benutzung des Fachbegriffs ursächlich für eine reduzierte Anzahl an korrekten Antworten sein. In zukünftigen Studien sollte dies berücksichtigt werden.

Bis zur Vorstellung in der Prämedikationsambulanz wurde bei 87 % (*n* = 170/196) der Befragten ein Blutbild erstellt, das bei 10 % (*n* = 20/196) der Befragten das Vorhandensein einer Anämie bestätigte. Aufgrund des Studiendesigns ist unklar, ob bei 91 % (*n* = 179/196) der Befragten keine Anämie vorliegt oder den Befragten die Diagnose einer Anämie (noch) nicht mitgeteilt wurde. In der Bevölkerung beträgt die Prävalenz einer Anämie 30 %, im chirurgischen Kontext kann sie sogar bis zu 35 % betragen [4]. Damit ist es unwahrscheinlich, dass nur 10 % (*n* = 19/196) der Befragten eine Anämie hatten. Oft sind die Ergebnisse des Blutbilds noch nicht vorhanden, wenn sich Patient:innen in der Prämedikationsambulanz vorstellen, sodass sie evtl. noch nichts von einer bestehenden Anämie wissen. Eine weitere Möglichkeit ist, dass den Patient:innen die Diagnose nicht mitgeteilt wurde, da sie vielleicht nur unter einer „milden Anämie“ leiden. Trotzdem kann auch eine „milde Anämie“ ein Risikofaktor sein [12].

In der vorliegenden Studie war ein Großteil der in der Prämedikationsambulanz befragten Teilnehmer:innen männlich (73,5 %). Dies könnte u. a. mit der hohen Anzahl befragter Patient:innen aus der Klinik für Herz- und Gefäßchirurgie sowie der Viszeral- und Thoraxchirurgie assoziiert sein. Der Anteil an männlichen Patient:innen aus diesen Fachabteilungen ist oft höher [13–16].

Trotz der immer noch hohen Prävalenz der Anämie im chirurgischen Kontext war 64,3 % (*n* = 126/196) der Befragten bewusst, dass eine Anämie zu höheren intra- und postoperativen Komplikationen führen kann. Dass sich zudem die postoperative Erholungsphase verlängern kann, kreuzten 59,2 % (*n* = 116/196) der Befragten an. Im Gegensatz dazu steht, dass eine präoperative Anämiekorrektur nur von 41,8 % (*n* = 82/196) angestrebt wurde. Vielen Patient:innen ist nicht bewusst, dass eine Korrektur der Anämie sinnvoll ist, wodurch viele diese vermutlich auch nicht aktiv präoperativ einfordern. Ein möglicher Grund könnte auch sein, dass viele der Patient:innen ihre Symptome nicht eindeutig einer Anämie zuordnen können.

Einige der Symptome einer Anämie, wie Müdigkeit oder geringere Leistungsfähigkeit, konnten 76 % (*n* = 149/196) der Befragten als diese definieren. Dagegen wurden Atemnot, Kopfschmerzen und Herzrhythmusstörungen nur von 21–40 % der Teilnehmer:innen mit einer Anämie assoziiert. Würden Patient:innen auch diese Symptome mit einer Anämie in Verbindung bringen, könnte dies einerseits dazu führen, dass Patient:innen aktiver eine Anämiebehandlung, v. a. auch präoperativ, einfordern und sich andererseits natürlich auch die Lebensqualität verbessert.

Die Ergebnisse der vorliegenden Studie zeigen, dass ein Wissensdefizit bei Patient:innen bezüglich der Anämie vorliegt. Es ist schwierig, eine Anämie zu behandeln, wenn Patient:innen ihre eigenen Symptome keiner Erkrankung zuordnen können. Es ist davon auszugehen, dass Patient:innen, die bereits unter einer Anämie leiden, ein fundierteres Vorwissen haben als diejenigen, die eine Erstdiagnose erhalten oder nicht unter einer Anämie leiden. Das spiegelt sich auch in der Wissenseinstufung wider. Der Großteil der Befragten (77 %) stufte das persönliche Wissen als „eher schlecht bis schlecht“ ein.

In der vorliegenden Studie konnte keine Korrelation zwischen den Bildungsabschlüssen und den korrekt gegebenen Antworten beobachtet werden.

Es gibt bereits vereinzelte Studien, die das Wissen über eine Anämie ermittelten [17]. Wiafe et al. befragten beispielsweise junge Erwachsene in Ghana über die Eisenmangelanämie und die Möglichkeiten, diese durch die Ernährung positiv zu beeinflussen. Die Mehrzahl der Jugendlichen wusste nicht, was eine Anämie ist, und wie man sie z. B. durch Ernährung positiv beeinflussen kann [18]. Im Gegensatz dazu steht die Studie von Zaini et al., die in Saudi-Arabien Männer und Frauen bezüglich einer Anämie befragten. Auffällig war, dass 25 % der Teilnehmenden nicht wussten, dass es einen Zusammenhang zwischen einer Anämie und einer Schwangerschaft gibt. Davon waren über 60 % Frauen. Weiterhin suggerieren die Studienergebnisse, dass sich die Inzidenz der Anämie durch noch mehr Aufklärungsarbeit reduzieren könnte [19].

Um die Risiken für die Patient:innen v. a. im operativen Setting zu minimieren, gibt es das Patient Blood Management, das sich auf 3 Säulen stützt: 1) präoperatives Anämiemanagement; 2) Minimierung von (intraoperativen) Blutverlusten; 3) leitliniengerechte Transfusion [20]. Dieses multimodale Konzept ist mittlerweile auch in der Hämotherapie-Querschnitts-Leitlinie von 2020 fest verankert [21]. Die internationale Konsensuskonferenz für das Anämiemanagement bei chirurgischen Patient:innen empfiehlt die Schulung von Patient:innen. Diese Schulung soll potenzielle Auswirkungen einer Anämie beinhalten und den Zusammenhang zwischen einer Anämie, einer Bluttransfusion und deren möglichen Folgen darlegen [22].

Die Gestaltung personalisierter Interventionen ist von wesentlicher Bedeutung, um die Beteiligung der Patient:innen an ihrer Behandlung aufrechtzuerhalten und sie zu ermutigen, eine aktive Rolle in ihrer eigenen Gesundheit und Gesundheitsfürsorge zu übernehmen. Informationen zu Ursachen, Behandlungsmöglichkeiten, möglichen Auswirkungen und Symptomen einer Anämie in Form von Flyern oder anderen Medien könnten das Selbstvertrauen und die Selbstbestimmung der Patient:innen fördern und zu einer besseren Gesundheit und Gesundheitsfürsorge beitragen.

## Fazit für die Praxis

Zusammenfassend konnte festgestellt werden, dass ein ausgeprägtes Wissensdefizit bei chirurgischen Patient:innen hinsichtlich einer Anämie vorliegt. Präoperative Anämien, insbesondere durch Eisen‑, Vitamin‑B_12_- oder Folsäuremangel bedingte Anämien, sind ernst zu nehmende und behandelbare Risikofaktoren. Daher sollten Patient:innen über die Ursachen und Risiken einer Anämie aufgeklärt werden.

Wir empfehlen …

- die Aufklärung der Patient:innen sowohl im klinischen sowie auch im ambulanten Bereich mithilfe von Informationsbroschüren, kurzen Wissensvideos oder auditiven Medien.
- die Schulung von Patient:innen bezüglich ihrer Symptome.
- die Schulung von Ärzt:innen bezüglich der Diagnosestellung und Behandlungsoptionen.
- die Erleichterung des Zugangs zu einer möglichen Anämiediagnostik und -behandlung.
- das Anämiescreening von Patient:innen, die eine Operation mit einer Transfusionswahrscheinlichkeit > 10 % erhalten.

## Supplementary Information

ESM 1_Aufstellung der Kliniken mit Indexoperationen

ESM 2_Fragebogen zur Anämieaufklärung

ESM 3_Patientenbezug zur Anämie

ESM 4_Wissenstand

## Data Availability

Die Daten werden auf begründeten Antrag zur Verfügung gestellt.
