# Supplementary material for: Current state of patient knowledge regarding the preoperative impact and causes of anemia
Source: Anaesthesiologie. 2025 Jan 31;74(2):81–8. [Article in German] doi: 10.1007/s00101-024-01498-y (PMC11836175; doi:10.1007/s00101-024-01498-y)
Supplement: Supplementary file 1 — ESM 1_Aufstellung der Kliniken mit Indexoperationen [file 101_2024_1498_MOESM1_ESM.pdf]

Zusatzmaterial zum Beitrag **„Aktueller Wissensstand von Patient:innen über den perioperativen Einfluss einer Anämie und ihrer Ursachen**“ von Mock J, Hof L, Dhein T et al. (2024) in *Die Anaesthesiologie.*

Beitrag und Zusatzmaterial stehen Ihnen auf www.springermedizin.de zur Verfügung. Bitte geben Sie dort den Beitragstitel in die Suche ein.

Indexoperationen mit einer Transfusionswahrscheinlichkeit >10%

Klinik für Allgemein-, Viszeral-, Transplantations- und Thoraxchirurgie

- Ösophagusresektion, Gastrektomie
- Rektumresektion, Pyloruserhaltende Pankreaskopfresektion
- Hemihepatektomie, Lebertransplantation, Nierentransplantation

Klinik für Gefäß- und Endovaskularchirurgie

- Periphere und zentrale Bypässe bei pAVK IIa und IIb
- Chirurgische Prothesen-Implantation bei Bauchaortenaneurysma

Klinik für Mund-, Kiefer- und Plastische Gesichtchirurgie

- Tumorlappen

Klinik für Unfall-, Hand- und Wiederherstellungschirurgie

- Endoprothesen Hüfte und Knie
- Lappenplastik
- Wirbelsäulenoperationen

Klinik für Herz-und Gefäßchirurgie

- Alle Eingriffe (Ausnahme: Schrittmacherimplantation und -explantation)

Klinik für Urologie

- Zystektomie
- Nephrektomie, Nierenteilresektion
- Prostatektomie
