# Supplementary material for: Current state of patient knowledge regarding the preoperative impact and causes of anemia
Source: Anaesthesiologie. 2025 Jan 31;74(2):81–8. [Article in German] doi: 10.1007/s00101-024-01498-y (PMC11836175; doi:10.1007/s00101-024-01498-y)
Supplement: Supplementary file 2 — ESM 2_Fragebogen zur Anämieaufklärung [file 101_2024_1498_MOESM2_ESM.pdf]

Zusatzmaterial zum Beitrag „Aktueller Wissensstand von Patient:innen über den perioperativen Einfluss einer Anämie und ihrer Ursachen“ von Mock J, Hof L, Dhein T et al. (2024) in *Die Anaesthesiologie.*

Beitrag und Zusatzmaterial stehen Ihnen auf www.springermedizin.de zur Verfügung. Bitte geben Sie dort den Beitragstitel in die Suche ein.

Fragebogen zur Anämieaufklärung

Liebe Teilnehmer*innen,

wir möchten die Qualität der Behandlung und der medizinischen Aufklärung am Universitätsklinikum Frankfurt stets verbessern. Mithilfe dieses Fragebogens helfen Sie uns dabei, festzustellen, ob Sie ausreichend informiert sind über Blutarmut/Anämie und zeigen uns, wie wir uns verbessern können.

Herzlichen Dank für Ihre Teilnahme!

1.) Wie alt sind Sie?

☐18 – 30 ☐31 – 40 ☐41 – 50 ☐51 – 60
☐61 – 70 ☐71 – 80 ☐> 80 Jahre ☐keine Angabe

2.) Welches Geschlecht haben Sie? ☐weiblich ☐männlich ☐divers ☐keine Angabe

3.) Was ist Ihr höchster Bildungsabschluss?

☐kein Abschluss ☐Hauptschulabschluss ☐Realschulabschluss
☐(Fach-)Abitur ☐Abgeschlossene Ausbildung ☐(Fach-) Hochschulabschluss
☐Ich weiß nicht ☐keine Angabe

☐Sonstiges: ______________________________

4.) In welcher Fachabteilung werden Sie behandelt?

☐ Klinik für Dermatologie
☐ Klinik für Frauenheilkunde und Geburtshilfe
☐ Klinik für Augenheilkunde
☐ Klinik für Allgemein-, Viszeral-, Transplantations- und Thoraxchirurgie
☐ Klinik für Herz- und Gefäßchirurgie
☐ Klinik für Mund-, Kiefer-, Plastische Gesichtschirurgie
☐ Klinik für Unfall-, Hand- und Wiederherstellungschirurgie
☐ Klinik für Urologie
☐ Klinik für Orthopädie (Friedrichsheim)
☐ Klinik für Neurologie und Neurochirurgie
☐ Ich weiß es nicht
☐ Sonstiges: ______________________________

5.) **Wurde bei Ihnen kürzlich hier im Krankenhaus ein Blutbild erstellt, bzw. Blut abgenommen?**

☐Ja ☐Nein ☐Ich weiß nicht

6.) **Wurde bei Ihnen schon einmal eine Blutarmut/ Anämie festgestellt?**

☐Ja ☐Nein ☐Ich weiß nicht

7.) **Leidet eines Ihrer Familienmitglieder oder einer Ihrer Freunde an einer Blutarmut/Anämie?**

☐Ja ☐Nein ☐Ich weiß nicht

8.) **Waren Sie bereits zur Untersuchung/Behandlung in unserer Anämieambulanz?**

☐Ja ☐Nein ☐Ich weiß nicht

Oder ist noch ein Besuch in unserer Anämieambulanz geplant (Laufzettel)?

☐Ja ☐Nein ☐Ich weiß nicht

9.) **Welche Aussagen zu Ursachen einer Blutarmut/Anämie sind richtig? Es können mehrere Aussagen richtig sein.**

	Richtig	Falsch	Weiß nicht
Eine Blutarmut/Anämie kann durch eine chronische Nierenerkrankung entstehen.	☐	☐	☐
Ein chronischer Blutverlust, z.B. im Rahmen einer Magenblutung oder Tumorerkrankung, kann zu einer Blutarmut/Anämie führen.	☐	☐	☐
Eine Blutarmut/Anämie wird durch die Ernährung beeinflusst.	☐	☐	☐
Eine der häufigsten Ursachen für eine Blutarmut/Anämie ist ein Eisenmangel.	☐	☐	☐
Eine Schilddrüsenerkrankung führt häufig zu einer Blutarmut/Anämie.	☐	☐	☐

10.) **Welche Aussagen zu der Untersuchung einer Blutarmut/Anämie sind richtig? Es können mehrere Aussagen richtig sein.**

	Richtig	Falsch	Weiß nicht
Bei einer Blutarmut/Anämie hat der Körper zu viele rote Blutzellen.	☐	☐	☐
Bei einer Blutarmut/Anämie ist der Hämoglobin (Hb)-Wert zu niedrig.	☐	☐	☐
Um eine Blutarmut/Anämie festzustellen, muss eine Urinprobe entnommen werden.	☐	☐	☐
Eine Blutarmut/Anämie kann im Krankenhaus und beim Hausarzt festgestellt werden.	☐	☐	☐

11.) Welche Symptome könnten aufgrund einer Blutarmut/Anämie auftreten? Es können mehrere Aussagen richtig sein.

	Richtig	Falsch	Weiß nicht
Geschwollene Knöchel	☐	☐	☐
Blasse Haut	☐	☐	☐
Müdigkeit, verringerte Leistungsfähigkeit	☐	☐	☐
Atemnot	☐	☐	☐
Kopfschmerzen	☐	☐	☐
Herzrhythmusstörungen	☐	☐	☐
Juckender Ausschlag	☐	☐	☐
Durchfall	☐	☐	☐

12.) Welchen Einfluss hat eine Blutarmut/Anämie auf eine Operation oder den Krankenhausaufenthalt? (Es können mehrere Aussagen richtig sein.)

	Richtig	Falsch	Weiß nicht
Eine Blutarmut/Anämie kann das Risiko für eine Bluttransfusion erhöhen.	☐	☐	☐
Eine Blutarmut/Anämie könnte die Erholungsphase nach einer Operation verlängern.	☐	☐	☐
Patienten mit Blutarmut/Anämie benötigen während der Operation mehr Narkosemedikamente.	☐	☐	☐
Es können mehr Komplikationen auftreten, wenn Patienten eine Anämie haben und während der Operation viel Blut verlieren.	☐	☐	☐
Falls möglich sollte eine Blutarmut/Anämie vor der Operation korrigiert werden.	☐	☐	☐

13.) Welche Aussagen bezüglich der Behandlung von Blutarmut/Anämie treffen zu? Es können mehrere Aussagen richtig sein.

	Richtig	Falsch	Weiß nicht
Zur Behandlung einer Eisenmangelanämie kann Eisen als Tablette eingenommen oder als Infusion verabreicht werden.	☐	☐	☐
Regelmäßiger Sport kann das Risiko für eine Blutarmut/Anämie senken.	☐	☐	☐
Eine Blutarmut/Anämie kann nur bei Frauen behandelt werden.	☐	☐	☐
Ernährungsbedingte Anämien, wie z.B. Eisenmangelanämien, Folsäure- oder Vitamin B12-Mangelanämien, sind gut behandelbar.	☐	☐	☐

14.) Welche Lebensmittel enthalten viel Eisen?

	Richtig	Falsch	Weiß nicht
Rotes Fleisch	☐	☐	☐
Äpfel	☐	☐	☐
Linsen	☐	☐	☐
Nüsse	☐	☐	☐

15.) Wie würden Sie Ihr Wissen über die Risiken und Behandlungsmöglichkeiten von Anämien einstufen?

☐gut ☐eher gut ☐eher schlecht ☐schlecht

16.) Wenn ich eine Anämie habe, möchte ich dazu mehr Informationen erhalten (Ursachen, Behandlungsmöglichkeiten, Risiken).

☐ Ja ☐ Nein ☐ Ich weiß nicht

17.) In welcher Sprache wünschen Sie sich mehr medizinische Informationen?

☐Deutsch ☐Englisch ☐Türkisch ☐Arabisch ☐Sonstiges: ____________________
