# Supplementary material for: Current state of patient knowledge regarding the preoperative impact and causes of anemia
Source: Anaesthesiologie. 2025 Jan 31;74(2):81–8. [Article in German] doi: 10.1007/s00101-024-01498-y (PMC11836175; doi:10.1007/s00101-024-01498-y)
Supplement: Supplementary file 3 — ESM 3_Patientenbezug zur Anämie [file 101_2024_1498_MOESM3_ESM.pdf]

Zusatzmaterial zum Beitrag „Aktueller Wissensstand von Patient:innen über den perioperativen Einfluss einer Anämie und ihrer Ursachen“ von Mock J, Hof L, Dhein T et al. (2024) in *Die Anaesthesiologie.*

Beitrag und Zusatzmaterial stehen Ihnen auf www.springermedizin.de zur Verfügung. Bitte geben Sie dort den Beitragstitel in die Suche ein.

Patientenbezug zur Anämie

Frage	Antwortmöglichkeit	n (%)
Wurde bei Ihnen kürzlich hier im Krankenhaus ein Blutbild erstellt, bzw. Blut abgenommen?	Ja	170 (86,7%)
	Nein	20 (10,2%)
	Ich weiß nicht	3 (1,5%)
	Keine Angabe	3 (1,5%)
Wurde bei Ihnen schon einmal eine Blutarmut/ Anämie festgestellt?	Ja	19 (9,7%)
	Nein	168 (85,7%)
	Ich weiß nicht	9 (4,6%)
	Keine Angabe	0 (0%)
Leidet eines Ihrer Familienmitglieder oder einer Ihrer Freunde an einer Blutarmut/Anämie?	Ja	6 (3,1%)
	Nein	164 (83,7%)
	Ich weiß nicht	26 (13,3%)
	Keine Angabe	0 (0%)
Waren Sie bereits zur Untersuchung/Behandlung in unserer Anämieambulanz?	Ja	10 (5,1%)
	Nein	179 (91,3%)
	Ich weiß nicht	6 (3,1%)
	Keine Angabe	1 (0,5%)
Oder ist noch ein Besuch in unserer Anämieambulanz geplant (Laufzettel)?	Ja	12 (6,1%)
	Nein	159 (81,1%)
	Ich weiß nicht	22 (11,2%)
	Keine Angabe	3 (1,5%)
