# Supplementary material for: Current state of patient knowledge regarding the preoperative impact and causes of anemia
Source: Anaesthesiologie. 2025 Jan 31;74(2):81–8. [Article in German] doi: 10.1007/s00101-024-01498-y (PMC11836175; doi:10.1007/s00101-024-01498-y)

Zusatzmaterial zum Beitrag „Aktueller Wissensstand von Patient:innen über den perioperativen Einfluss einer Anämie und ihrer Ursachen“ von Mock J, Hof L, Dhein T et al. (2024) in *Die Anaesthesiologie.*

Beitrag und Zusatzmaterial stehen Ihnen auf www.springermedizin.de zur Verfügung. Bitte geben Sie dort den Beitragstitel in die Suche ein.

Abb I - Detaillierte Übersicht zu „Einfluss der Ernährung“

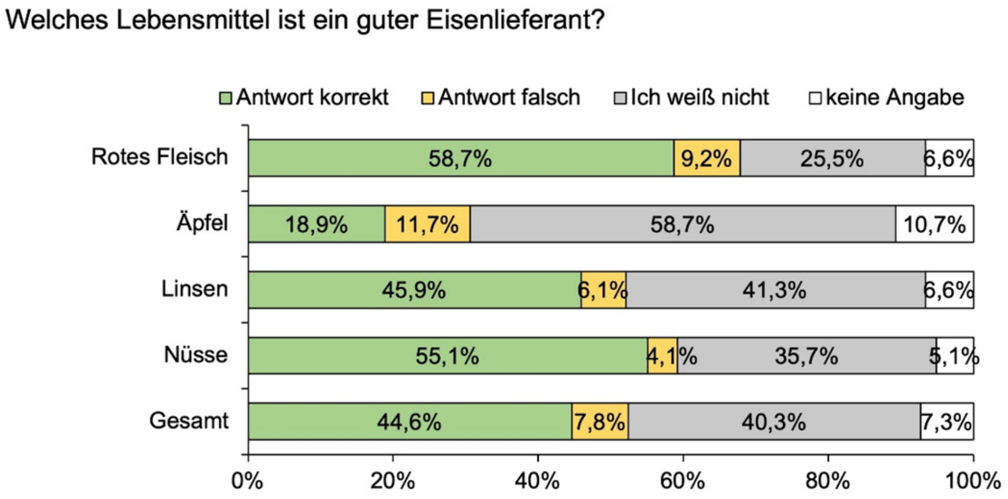

Abb II - Detaillierte Übersicht über die Kategorien „Behandlung einer Anämie“

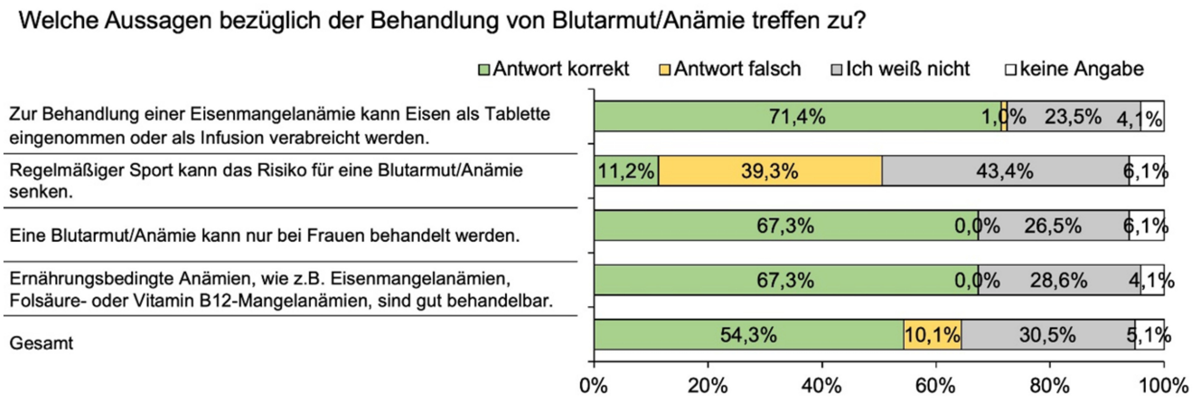

Supplement: Supplementary file 4 — ESM 4_Wissenstand [file 101_2024_1498_MOESM4_ESM.pdf]
